# A novel human STAT3 mutation presents with autoimmunity involving Th17 hyperactivation

**DOI:** 10.18632/oncotarget.5042

**Published:** 2015-07-30

**Authors:** Judith Wienke, Willemijn Janssen, Rianne Scholman, Hilde Spits, Marielle van Gijn, Marianne Boes, Joris van Montfrans, Nicolette Moes, Sytze de Roock

**Affiliations:** ^1^ Paediatric Immunology, Laboratory of Translational Immunology LTI, University Medical Center Utrecht, Utrecht, The Netherlands; ^2^ Multiplex core facility, Laboratory of Translational Immunology LTI, University Medical Center Utrecht, Utrecht, The Netherlands; ^3^ Department of Medical Genetics, University Medical Center Utrecht, Utrecht, The Netherlands; ^4^ Department of Paediatric Gastroenterology, Hepatology and Nutrition, University Medical Center Groningen, Groningen, The Netherlands

**Keywords:** autoimmunity, STAT3, Th17, IL-17, IL-21, Immunology and Microbiology Section, Immune response, Immunity

## Abstract

Mutations in STAT3 have recently been shown to cause autoimmune diseases through increased lymphoproliferation. We describe a novel Pro471Arg STAT3 mutation in a patient with multiple autoimmune diseases, causing hyperactivation of the Th17 pathway. We show that IL-17 production by primary T cells was enhanced and could not be further increased by IL-6, while IL-10 reduced Th17 cell numbers. Moreover, specific inhibition of STAT3 activation resulted in diminished IL-17 production. We show that the Pro471Arg STAT3 mutation yields both increased levels of IgA and IgG, probably due to high IL-21 levels. When remission was reached through medical intervention, IL-17 levels normalized and the clinical symptoms improved, supporting the idea that STAT3 gain-of-function mutations can cause hyperactivation of the Th17 pathway and thereby contribute to autoimmunity.

## INTRODUCTION

Three recently published studies show that STAT3 mutations can underlie autoimmune pathology [1-3]. Although these studies provide pathophysiological explanations as causes for the disease phenotype for several STAT3 mutations, the disease mechanisms caused by STAT3 mutations are not yet fully understood. STAT3 has a role in the development of Th17 cells, which have been associated with several autoimmune diseases [4]. We here provide experimental immunological support in an exploratory study that increased Th17 activity can contribute to autoimmune pathology in patients with STAT3 mutations.

## RESULTS

A 17 year old female was referred for immune-diagnostics because of multi-organ autoimmune disease that was present since early childhood. Immune dysregulation polyendocrinopathy enteropathy X-linked syndrome (IPEX) or IPEX-like syndrome were excluded by showing normal regulatory T cell (Treg) number and function (Supplemental Figure 1A, Supplemental patient description and Supplemental Tables 1 & 2). Screening of primary immunodeficiency related genes [5] showed a *de novo* heterozygous mutation in STAT3 (c.1412C>G p.(Pro471Arg), NM_139276.2) with potential pathological consequences (see Supplemental Figure 1B & 1C). The patient and her parents gave written informed consent for this study and blood was sampled during active disease and remission on Rapamycin treatment.

**Figure 1:**
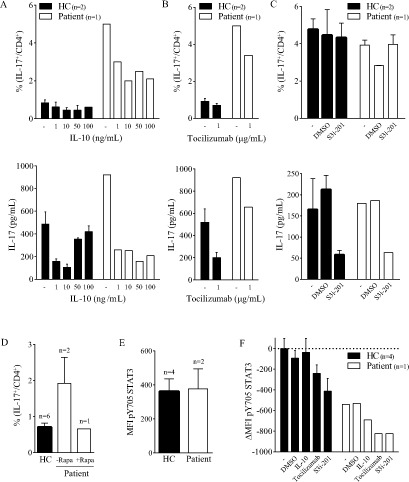
STAT3 Pro471Arg mutation causes hyperactive Th17 pathway and aberrant B cell phenotype **A.** Blood plasma levels of IL-6, IL-17 and IL-21. **B.** IL-6, IL-17 and IL-21 production by *ex vivo* PMA/Ionomycin activated PBMC. **C.** Percentage of circulating IL-17^+^ CD4^+^ T cells. **D.** Increasing concentrations of IL-6 were added to PBMC cultures with plate bound anti-CD3 (‘-’); the intracellular expression of IL-17 and RORγt in CD4^+^ T cells was measured by flow cytometry (left) and the amount of IL-17 produced was determined in the supernatant after four days of culture (right). **E**. **F**. B cell phenotype; percentage of circulating plasmablasts (**E)** and IgA and IgG expression (**F)**. **G.** Increasing concentrations of IL-21 were added to PBMC cultures with plate bound anti-CD3 (‘-’); surface expression of IgA (left) and IgG (right) was measured by flow cytometry (*n* = 3). *Error bars represent SEM.*

Considering that IL-6 mediated STAT3 activation drives Th17 cell development [6] we tested components of this pathway in blood plasma and peripheral blood mononuclear cells. First, we explored circulating cytokines involved in the Th17 pathway by multiplex analysis of plasma obtained during active disease. IL-6, IL-17 and IL-21 were elevated in the patient compared to healthy donors (Figure 1A). In accordance with this, PMA/ionomycin activated PBMC of the patient showed increased numbers of IL-17 producing cells and increased RORγt expression within the CD4^+^ T cell compartment (Figure 1B). In contrast, IL-21 and IL-6 production by PBMC was reduced, possibly as a compensatory mechanism for already high plasma levels (Figure 1C). To further assess the nature of this elevated Th17 cell activity we cultured PBMC with increasing concentrations of IL-6. The number of Th17 cells was already markedly elevated in the patient cell culture, irrespective of IL-6 stimulation (Figure 1D). Addition of IL-6 did not result in more Th17 cells, but showed a dose-dependent increase of IL-17 in the culture supernatants of both the healthy donors and patient. In contrast, STAT3 activating cytokine IL-23 did not have an IL-17 inducing effect on patient cells (not shown). These results show a contribution of the Pro471Arg STAT3 mutation to a persistent hyperactivation of the Th17 response independent of IL-6 signaling.

Next to the aberrant T cell phenotype we observed B cell abnormalities in the patient, characterized by an increased presence of plasmablasts and elevated IgA and IgG surface expression *ex vivo* (Figure 1E-1F). This observation correlated with elevated concentrations of IgA and IgG in patient plasma as compared to healthy donor values (see Supplemental Figure 2). It is well known that B cell class switching, plasma cell formation and immunoglobulin production depend amongst others on IL-21 [7] which is elevated in our patient. To investigate whether Th17 cell effector cytokines could account for the observed B cell abnormalities, we cultured healthy donor PBMC with IL-17 and IL-21. IL-21 caused a dose-dependent increase of class switched memory B cells and surface IgA and IgG expression (Figure 1G), as has been shown before [8]. IL-17 had no effect on B cell maturation and immunoglobulin expression (data not shown). Thus the STAT3 dependent high IL-21 levels observed in the patient during active disease may contribute to the hypergammaglobulinemia phenotype of the patient.

**Figure 2:**
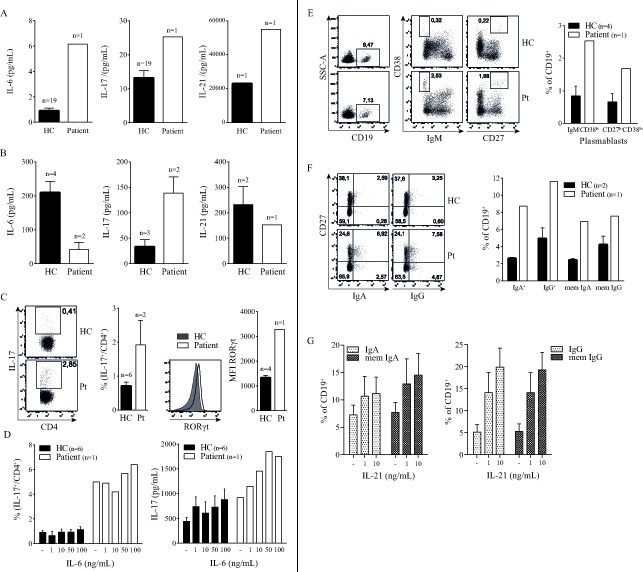
Th17 hyperactivation can be dampened PBMC were cultured with plate bound anti-CD3 (‘-’) with or without IL-10 (**A)**, tocilizumab (**B)** or S3i-201 (**C)** for 4 days; the percentage of IL-17^+^ CD4^+^ T cells was measured by flow cytometry (upper panel) and the amount of IL-17 produced in the supernatant was determined by multiplex immunoassay (lower panel). **D.** Percentage of IL-17+ CD4+ T cells in the patient during and after treatment with Rapamycin (HC and -Rapa same data as Figure 1C) **E.** MFI of *ex vivo* pY705 STAT3 in total T cells. **F.** ΔMFI (relative to HC with anti-CD3 only) of pY705 STAT3 after 4 day culture as described above (IL-10: 10 μg/mL). Negative values indicate decrease in MFI. *Error bars represent SEM.*

To investigate whether the increased Th17 activity in the patient could be influenced by agents that are known to modulate the STAT3-Th17 pathway, we cultured PBMC with IL-10, IL-6 receptor blocking antibody tocilizumab and STAT3 inhibitor S3i-201. Increasing concentrations of IL-10 did not clearly affect healthy donor Th17 cells, but in patient cell cultures the number of Th17 cells and the amount of IL-17 produced were decreased (Figure 2A). Tocilizumab reduced the Th17 activity in healthy donors and the patient (Figure 2B), whereas S3i-201 did not have an effect on Th17 numbers, but only reduced the IL-17 production (Figure 2C). We tested the Th17 response again when the patient showed reduced disease activity during treatment with Rapamycin. Indeed, concomitant with an increase in Treg numbers (not shown), Rapamycin treatment caused a reduction in the number of IL-17 producing T cells after PMA/Ionomycin stimulation (Figure 2D). These results indicate that even though Th17 activity is markedly increased in the patient, it can be dampened by (in)direct inhibition of the STAT3-Th17 axis, *in vitro* as well as *in vivo*.

To formally address whether an altered phosphorylation state of the STAT3 protein may contribute to the patient's phenotype, we analyzed the phosphorylation of the tyrosine residue 705 (pY705) of STAT3 in fresh cells and upon culture with anti-CD3 with or without IL-10, tocilizumab or S3i-201. The Y705 phosphorylation status was comparable between the patient and healthy donor cells (Figure 2E). After 4-day culture, STAT3 Y705 phosphorylation was even lower in patient cells. Addition of tocilizumab or S3i-201 did reduce the phosphorylation in both healthy donor and patient cells (Figure 2F). Thus, constitutive phosphorylation of the STAT3 Y705 tyrosine does not explain the observed phenotype.

## DISCUSSION

In summary, we here show in primary patient material that the Pro471Arg STAT3 mutation correlates with a highly activated Th17 pathway and contributes to autoimmune pathology and activated B cells. The elevated IL-21 levels (which could be produced by Th17 cells but also by NKT and T follicular helper cells) as found in the patient plasma likely supports class switching and could thereby contribute to the observed hypergammaglobulinemia. The clinical characteristics of the patient described here and the patients described elsewhere [1-3] clearly diverge from hyper-IgE syndromes that are associated with STAT3 mutations [9]. The diversity in clinical manifestations of STAT3 mutations suggests that mutations in this gene might underlie more autoimmune disorders, specifically when disease onset occurs at early age. While we concede that the Th17 pathway may not account for all these phenotypes and most data are obtained from a limited number of samples, the immunological data provided are suggestive of the Th17 pathway contributing to more autoimmune disorders than currently recognized. We therefore suggest that sequencing of STAT3 should be considered in more patients with severe autoimmunity, and specified treatment options focusing on the Th17 pathway like tocilizumab should be further explored for these patients.

## MATERIALS AND METHODS

Patient material was collected once during active disease and once during disease remission on Rapamycin treatment. Plasma and peripheral blood mononuclear cells (PBMC) of patient and healthy controls were stored at −150°C for later use in experiments. Cytokine concentrations were measured in plasma and culture supernatants by multiplex immunoassay as described elsewhere [10]. PBMC were activated with 20 ng/ml phorbol 12-myristate 13-acetate (PMA) and 1 μg/ml ionomycin for 4 hours to measure cytokine production in supernatant or by 1 μg/ml anti-CD3 (okt3, eBioscience, San Diego, USA) and cultured for four days with or without recombinant IL-6 (Miltenyi, Bergisch Gladbach, Germany) or IL-10 (BD Bioscience, San Jose, USA). Monensin (Golgistop, BD Bioscience) was added during the last 3.5 h PMA/Ionomycin stimulation for flow cytometric analysis of intracellular cytokine expression. STAT3 activity was inhibited by addition of 50 μM S3I-201 (Santa Cruz Biotechnology, Heidelberg, Germany) and phosphorylation investigated with anti-pY705 STAT3 (Clone 4/P-STAT3 BD Bioscience). For analysis of B cell differentiation, PBMC were cultured for 5 days with plate bound anti-CD3 and rhIL-21 (1 and 10 ng/ml; Immunotools) or rhIL-17 (1 and 10 ng/ml; Immunotools). Suppression assays were conducted with Treg inspector beads (Miltenyi) and CD4^+^CD25^+^CD127^−^ sorted Treg.

## SUPPLEMENTARY MATERIAL TABLES AND FIGURES



## Abbreviations

IL: Interleukin
IPEX: Immune dysregulation polyendocrinopathy enteropathy X-linked
PBMC: Peripheral blood mononuclear cells
PMA: Phorbol 12-myristate 13-acetate
RORγT: RAR related orphan receptor gamma T
STAT3: Signal transducer and activator of transcription 3
Teff: T effector cell
Th: T-helper
Treg: Regulatory T cell
