# Supplementary material for: A novel human STAT3 mutation presents with autoimmunity involving Th17 hyperactivation
Source: Oncotarget. 2015 Jul 30;6(24):20037–42. doi: 10.18632/oncotarget.5042 (PMC4652985; doi:10.18632/oncotarget.5042)
Supplement: Supplementary file 1 [file oncotarget-06-20037-s001.pdf]

A novel human STAT3 mutation presents with autoimmunity involving Th17 hyperactivation

Supplementary Material

Patient description

Patient History

17 year old female born full term as a first child to non-consanguineous parents presented with autoimmune symptoms in the first months of life, starting with alopecia.

At the age of 8 months she presented first signs of autoimmune enteropathy (positive anti-enterocyte antibodies) for which she was treated with parenteral nutrition and tacrolimus. She was also diagnosed with celiac disease based on antibodies and histology (Marsh 3b/c). In the following years the enteropathy was controlled with gluten free diet and azathioprine.

At the age of 10 years she developed autoimmune hypothyroidism. At age 14 she received a liver transplant due to acute liver failure as a result of autoimmune hepatitis (ANA+, anti-smooth muscle intermediate).

After transplantation and under the immunosuppressive treatment of prednisone, tacrolimus and azathioprine, her autoimmune disease remained stable for several years.

However, recently, she had a relapse of her autoimmune enteropathy, making it necessary to increase prednisone and tacrolimus dosage and eventually to add Rapamycin.

The blood sampling for this study was performed prior to the addition of Rapamycin, unless otherwise indicated.

Supplementary Table 1. Clinical Characteristics.

**Patient characteristics**	**Clinical data**
STAT3 mutation	c.1412C>G p.(Pro471Arg)
Sex	Female
Age	17 y
Birthweight (SD)	-1.5
Growth (SD)	-2.5
Puberty	Normal
Infection Susceptibility	Recurrent *Herpes zoster* infections under immunosuppression

Supplementary Table 2. Rapamycin treatment intervals

Aug 2013	Feb 2014	May 2014	Nov 2014	Jan 2015
Start	Non compliance, undetectable blood levels	Sufficient blood levels	Stop	Restart

Supplementary Table 3. Symptoms and age of onset.

Organ system	Symptoms	Age of Onset
Skin	Alopecia	6m
	Eczema	6m
Gastrointestinal	Auto-immune Enteropathy	8m
	Celiac Disease	8y
	Hepatitis	14y
Endocrine	Hypothyreosis	10y
Pulmonary	Lower Diffusion Capacity	16y
Cardiovascular	No symptoms	-
Renal & Urinary	No symptoms	-
Hematological	No symptoms	-

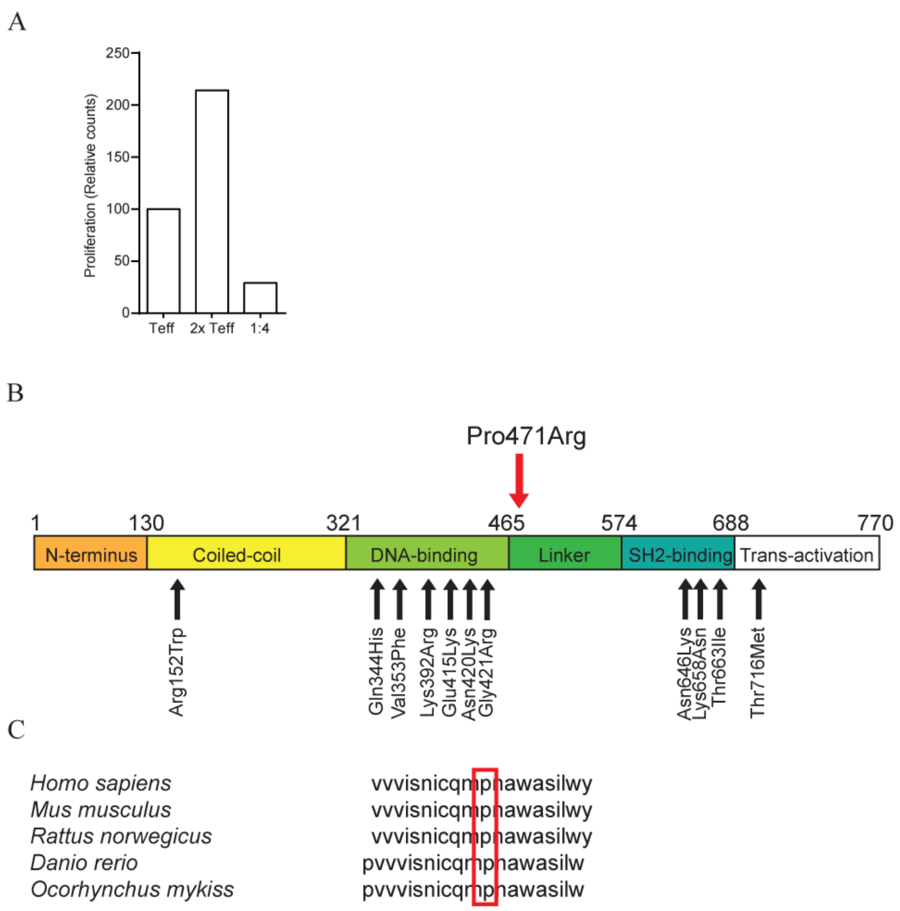

Supplementary Fig 1. Treg suppression assay and described mutations in and evolutionary conservation of the STAT3 gene. (A) Suppression of bead-activated Teff cells by patient derived Treg. ^{3}H-counts of effector (CD4) T cells alone were set to 100% and compared to 2x higher numbers of Teff per well, or Teff with 1:4 Treg added. (B) Location of the Pro471Arg mutation in the STAT3 gene (red arrow) and already described gain-of-function mutations (black arrows) and (C) conservation of the amino acid order throughout species.

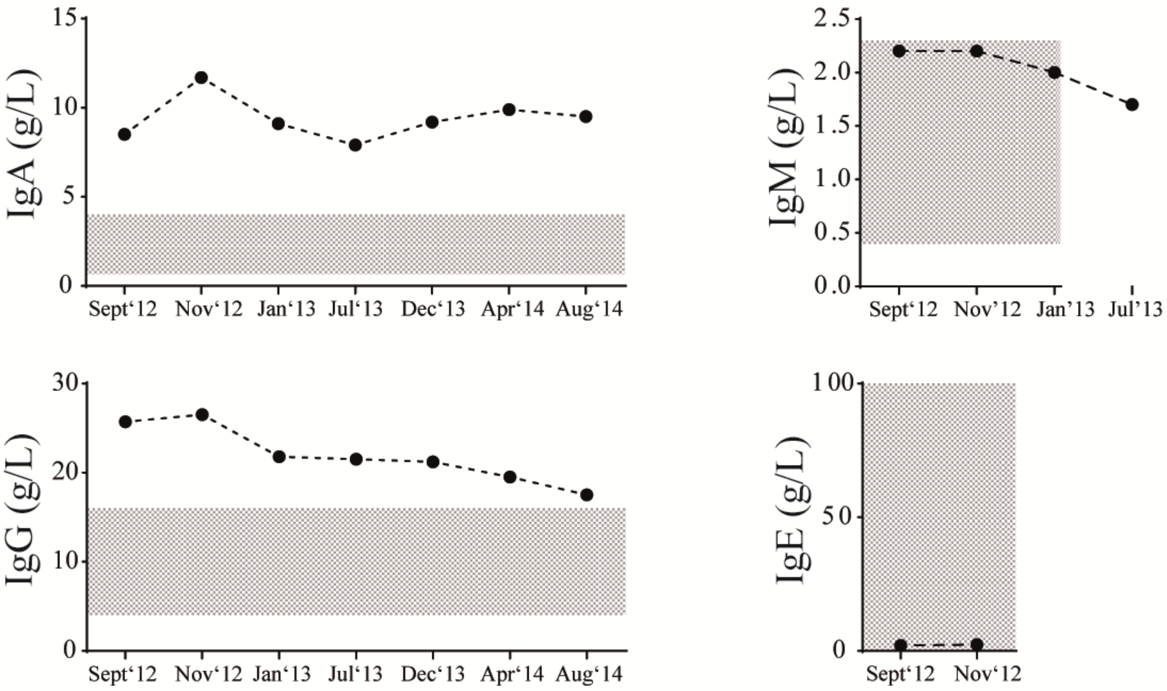

Supplementary Fig 2. Immunoglobulin levels in patient blood plasma.

Levels measured at several time-points, compared to standard healthy donor values (shaded area).
